# Multiregional-Based Magnetic Resonance Imaging Radiomics Combined With Clinical Data Improves Efficacy in Predicting Lymph Node Metastasis of Rectal Cancer

**DOI:** 10.3389/fonc.2020.585767

**Published:** 2021-02-18

**Authors:** Xiangchun Liu, Qi Yang, Chunyu Zhang, Jianqing Sun, Kan He, Yunming Xie, Yiying Zhang, Yu Fu, Huimao Zhang

**Affiliations:** ^1^ Department of Radiology, The First Hospital of Jilin University, Changchun, China; ^2^ Clinical Science Team, Philips Investment Co. Ltd., Shanghai, China

**Keywords:** rectal cancer, magnetic resonance imaging (MRI), machine learning, radiomics, lymph nodes

## Abstract

**Objective:**

To develop and validate a multiregional-based magnetic resonance imaging (MRI) radiomics model and combine it with clinical data for individual preoperative prediction of lymph node (LN) metastasis in rectal cancer patients.

**Methods:**

186 rectal adenocarcinoma patients from our retrospective study cohort were randomly selected as the training (n = 123) and testing cohorts (n = 63). Spearman’s rank correlation coefficient and the least absolute shrinkage and selection operator were used for feature selection and dimensionality reduction. Five support vector machine (SVM) classification models were built using selected clinical and semantic variables, single-regional radiomics features, multiregional radiomics features, and combinations, for predicting LN metastasis in rectal cancer. The performance of the five SVM models was evaluated *via* the area under the receiver operator characteristic curve (AUC), accuracy, sensitivity, and specificity in the testing cohort. Differences in the AUCs among the five models were compared using DeLong’s test.

**Results:**

The clinical, single-regional radiomics and multiregional radiomics models showed moderate predictive performance and diagnostic accuracy in predicting LN metastasis with an AUC of 0.725, 0.702, and 0.736, respectively. A model with improved performance was created by combining clinical data with single-regional radiomics features (AUC = 0.827, (95% CI, 0.711–0.911), *P* = 0.016). Incorporating clinical data with multiregional radiomics features also improved the performance (AUC = 0.832 (95% CI, 0.717–0.915), *P* = 0.015).

**Conclusion:**

Multiregional-based MRI radiomics combined with clinical data can improve efficacy in predicting LN metastasis and could be a useful tool to guide surgical decision-making in patients with rectal cancer.

## Introduction

Colorectal cancer was the third most common type of malignant tumor and the second leading cause of cancer death in the world in 2018 (1). Nearly one-third of colorectal tumors are located in the rectum (2). Lymph node (LN) status plays a vital role in determining whether to perform adjuvant therapy or additional surgical resection (2–6). Therefore, accurate preoperative assessment of LN status or assessment of the N stages of regional LNs in rectal cancer patients *via* medical imaging is essential for precise individualized decision making and patient prognosis (2, 6, 7). However, preoperative LN staging in rectal cancer patients remains a challenge for radiologists (4).

Magnetic resonance imaging (MRI) is considered the most accurate method to assess the primary staging of rectal cancer (2). However, MRI, computed tomography (CT) and endorectal ultrasound cannot reliably evaluate LN metastasis (2, 4, 8). All diagnostic clues rely heavily on the size, shape, and margins of LNs, but these semantic characteristics alone are insufficient to reliably distinguish malignant from benign LNs in rectal cancer patients (2, 4, 5, 9).

Unlike traditional image evaluation methods, radiomics is an emerging and effective method for quantitatively analyzing the classification and prognosis of diseases using medical imaging (10). From standard-of-care medical images, data can be extracted *via* high-throughput mining of quantitative image features, which are undetectable by the naked eye, and applied within clinical-decision support systems (9–13); radiomics plays an important role in early diagnosis, treatment evaluation, and tumor prognosis prediction, ultimately aiding in the achievement of precision medicine (11, 14, 15).

In previous studies, a CT radiomics signature-based nomogram (16) and T2-weighted histogram of the primary tumor (17) have been applied and shown to successfully discriminate LN metastasis in colorectal- and rectal cancer patients. MRI can provide multiparameter images different from those obtained by CT, so it is of interest whether there exists an association between LN status and multiregional radiomics features of multiparametric MR images in rectal cancer patients. To the best of our knowledge, the topic has not been previously studied.

This study aimed to develop and validate a multiregional radiomics prediction model based on MRI and combine it with clinical-semantic data for the individualized preoperative prediction of LN metastasis in rectal cancer patients. This would allow clinicians to make personalized treatment plans.

## Materials and Methods

This retrospective study was approved by the ethics committee of the First Hospital of Jilin University, and the requirement for informed consent was waived.

### Patients

**Figure 1:**
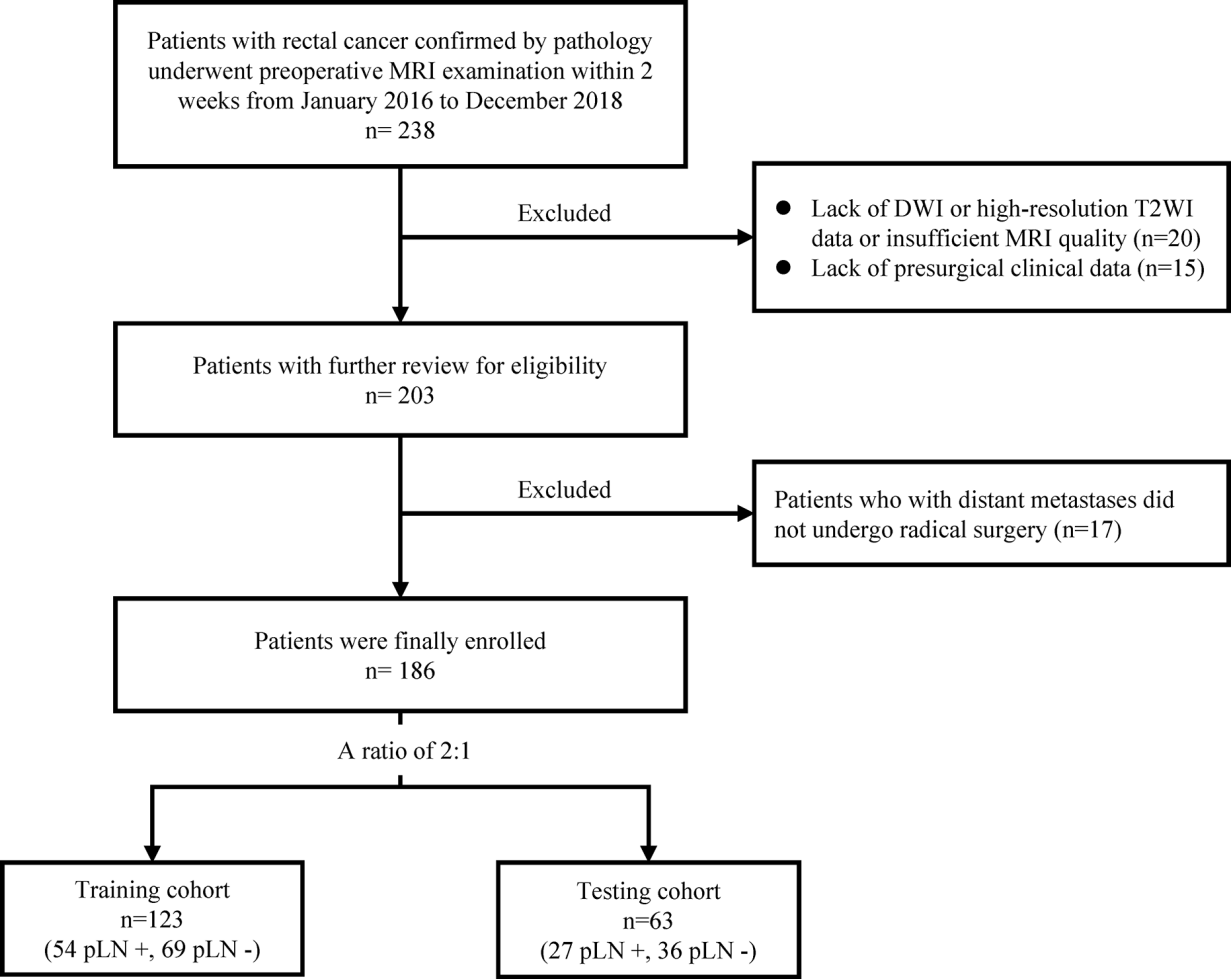
The process of patient selection. MRI, magnetic resonance imaging; pLN+, pathological lymph node positive; pLN−, pathological lymph node negative.

The data of 238 consecutive patients with rectal cancer from January 2016 to December 2018 were initially retrieved from the institutional database. The inclusion criteria were as follows: (i) rectal MRI examination was performed within the 2 weeks before surgery; (ii) the distal border of the tumor was ≤15 cm above the anal verge based on colonoscopy; (iii) subsequent radical surgical resection was performed; (iv) postoperative histopathological examination confirmed rectal adenocarcinoma; and (v) all LNs were assessed. The exclusion criteria were as follows: (i) distant metastases; (ii) not undergoing surgery at our hospital or lack of diffusion-weighted imaging (DWI) or high-resolution T2-weighted imaging (T2WI) data; (iii) insufficient MRI quality to obtain measurements (*e.g.*, owing to motion artifacts); and (iv) lack of presurgical carcinoembryonic antigen (CEA) and carbohydrate antigen 19-9 (CA19-9) data. A total of 186 patients met the criteria and were included in this study; they were divided randomly into a training cohort (n = 123) and a testing cohort (n = 63) at a ratio of 2:1. The process of patient selection is summarized in **Figure 1**.

Baseline clinicopathologic data, including age, gender, and levels of CA19-9 and CEA, were derived from medical records. Laboratory analyses of CEA and CA19-9 were conducted within 1 week before surgery. The threshold value for CEA was 5 ng/ml and that for CA19-9 was 39 U/ml, according to the clinically normal range.

### Radiomics Workflow

**Figure 2:**
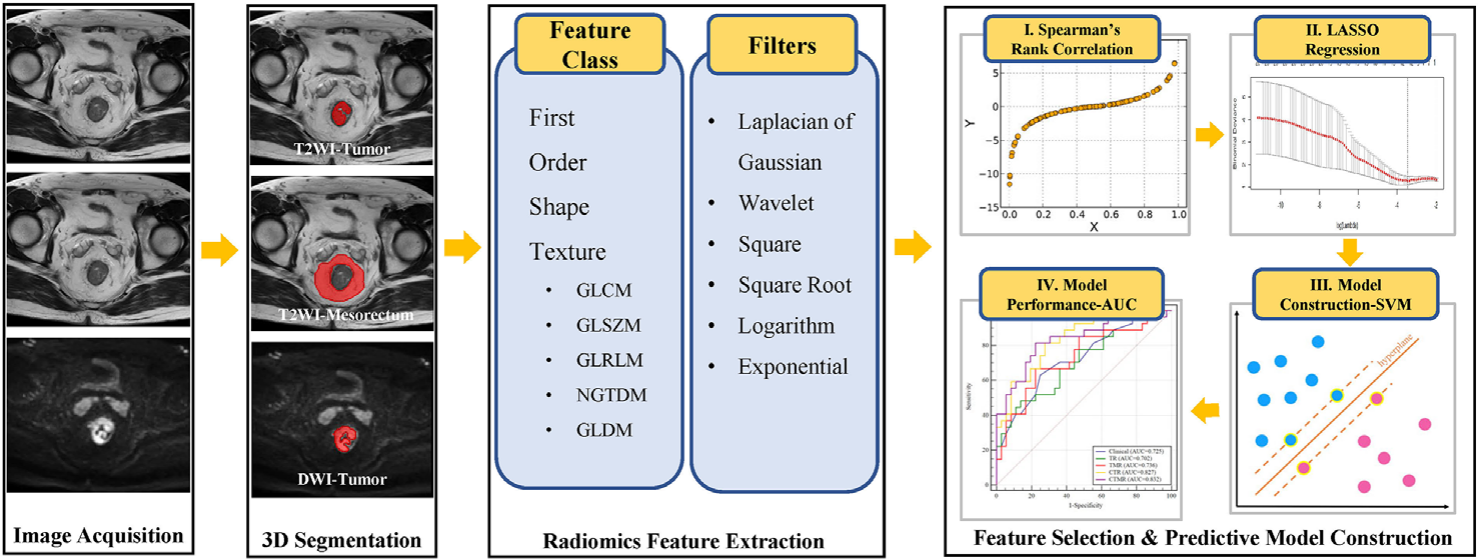
The workflow of radiomics in this study. T2WI, T2-weighted imaging; DWI, diffusion-weighted imaging; GLCM, Gray Level Cooccurence Matrix; GLSZM, Gray Level Size Zone Matrix; GLRLM, Gray Level Run Length Matrix; NGTDM, Neighboring Gray Tone Difference Matrix; GLDM, Gray Level Dependence Matrix; LASSO, least absolute shrinkage and selection operator; AUC, area under the curve; SVM, support vector machine.

The radiomics workflow is illustrated in **Figure 2** and includes (1) medical image acquisition, (2) tumor segmentation, (3) radiomics feature extraction, and (4) feature selection and predictive model construction (described in detail in the *Statistical Analysis* section).

### Medical Image Acquisition

All rectal MRIs were performed using a 3.0T MR scanner (Philips Ingenia, the Netherlands) with the patient in the supine position. To reduce colonic motility, 20 mg of anisodamine was injected intramuscularly 30 min before the MRI scan. All patients underwent the standard rectal MRI protocol including sagittal, axial, oblique axial, and coronal T2WI and DWI. DWI images were obtained with two b-factors (0 and 1,000 s/mm^2^), a repetition time (TR) of 2,800 ms, an echo time (TE) of 70 ms, a field of view (FOV) of 340 mm× 340 mm, a matrix of 256 × 256, a thickness of 4.0 mm, and a gap of 1.0 mm. Apparent diffusion coefficient maps were generated automatically and included both b-values. High-resolution T2WI images were obtained using turbo spin-echo with a TR of 3,500 ms, a TE of 100 ms, a FOV of 180 mm× 180 mm, an echo train length of 24, a matrix of 288 × 256, a thickness of 3.0 mm, and a gap of 0.3 mm.

### Semantic and Pathological Evaluation

Two radiologists with 3 years and 8 years of experience in rectal cancer MRI interpretation who were blinded to the histopathology results evaluated the MR images.

Conventional semantic evaluation indicators included MRI-reported LN status, which were performed using the qualitative criteria of the LNs according to the updated recommendations from the 2016 European Society of Gastrointestinal and Abdominal Radiology (ESGAR) consensus meeting (18). AN LN with a short-diameter of ≥9 mm is considered metastatic. An LN with a short diameter of 5–8 mm and at the same time satisfying any two of the following three items is considered metastatic: the edge of the LN is not smooth, the signal inside the LN is not uniform, and the LN is round. An LN with a short-diameter of <5 mm LN meeting all three of the above items is considered metastatic. The location of the primary tumor was measured on the approximate luminal center of the rectum on the sagittal T2WI sequence and categorized as lower (0–5 cm from the anal verge to the lowest edge of the tumor), middle (5.1–10 cm from the anal verge to the lowest edge of the tumor), or higher (10.1–15 cm from the anal verge to the lowest edge of the tumor) (5, 19). The tumor length (measured on the sagittal T2WI), tumor thickness (measured on the oblique axis T2WI), extramural depth of invasion (measured on the oblique axis T2WI), invasion of mesorectal fascia (MRF; >1 mm was diagnosed as negative and ≤1 mm diagnosed as positive), maximum LN short diameter (measured on the axis T2WI) were also evaluated. Cases of disagreement on the evaluation of semantic features were resolved through discussion between the two radiologists.

The pathological LN status of each patient was recorded following the histopathological reports.

### Tumor Segmentation

All MRI scans were retrieved from the picture archiving and communication system (Agfa) for tumor masking and image feature extraction.

One radiologist who was blinded to the histopathology results segmented the volumes of interest (VOIs) on high-spatial resolution T2WI and DWI images using IntelliSpace Discovery (Philips, Best, the Netherlands). For each patient, three VOIs were defined as follows: (i) the volume of the whole primary tumor on T2WI, which was manually drawn along the contour of the tumor on each slice; (ii) the volume of the whole primary tumor on DWI (b-value of 1,000 s/mm^2^), manually drawn on each slice on the high signal intensity region; and (iii) the volume of the peritumoral mesorectum on un-fat-suppressed T2WI, drawn along the MRF and the outer edge of the tumor and rectal wall, respectively, retaining the area between the two circles.

To assess intra-reader and inter-reader reproducibility, randomly selected T2WI images of 20 cases was segmented again by the same radiologist a month following the same procedure, as well as by another radiologist with 8 years’ experience in interpreting pelvic MRI.

### Radiomics Feature Extraction

For each patient, we used three different VOIs for radiomics feature calculation. Radiomics feature extraction was implemented using a Philips Radiomics Tool (Philips Healthcare, China); the core feature calculation was based on pyRadiomics (20).

For each VOI, a total of 1,653 three-dimensional (3D) radiomic features, including direct features, indirect features, Wavelet transform features, and Laplacian of Gaussian filtered features, were extracted. The types, introduction of extracted features, and the number of each type are shown in **Supplementary Table 1**. For each patient, we integrated all 4,959 radiomics features from three VOIs.

### Statistical Analysis

The statistical analysis of clinicopathological features and semantic indicators were performed with SPSS software (version 22.0, Chicago, IL, USA). The lasso algorithm and SVM model construction were implemented with the scikit-learn package in Python(3.7). *P <*0.05 was considered statistically significant using two-tailed testing.

#### Demographic Comparison of the Training and Testing Cohorts

The differences in continuous variables, including age, tumor length, tumor thickness, extramural depth of invasion, and maximum LN short diameter, between the training and testing cohorts were compared using a two-sample t-test or Mann–Whitney U test, according to the normality of data distribution tested using the Kolmogorov–Smirnov method. Chi-square or Fisher’s exact tests were used, as appropriate, to compare differences (including LN prevalence) in categorical variables (gender, location of the primary tumor, levels of CEA and CA19-9, invasion of MRF and MRI-reported LN status). The same statistical analysis was applied to assess differences in the characteristics between patients with pLN− (pathological N0 stage) and pLN+ (pathological N1–N2 stage) in the two cohorts.

#### Inter- and Intra-Observer Reproducibility of Tumor Segmentation

Dice similarity coefficient (DSC) was calculated to evaluate the inter-and intra-observer agreements of tumor segmentation. DSC greater than 0.75 indicates good agreement.

#### Feature Selection

First, all features (including baseline clinicopathological data, semantic indicators, and radiomics features) were normalized using the Min–Max scaling algorithm, as shown below:

$${\text{X}}_{\text{normal}}=\frac{\text{X}-{\text{X}}_{\text{min}}}{{\text{X}}_{\text{max}}-{\text{X}}_{\text{min}}}$$

Next, Spearman’s rank correlation coefficient analysis between each feature and label was performed. Features with a coefficient lower than an absolute value of 0.2 or *P* values greater than 0.05 were removed due to the low correlation between these features and the pathological labels. We then used the least absolute shrinkage and selection operator (LASSO) algorithm for dimensionality reduction (21).

#### Model Training and Validation

Five support vector machine (SVM) classification models were built using selected clinical and semantic features, single-regional radiomics features, multiregional radiomics features, and combinations thereof. The clinical model was developed based on selected clinical and semantic factors. The radiomics model of the tumor (TR) was developed based on selected radiomics features of two VOIs of the primary tumors. The radiomics model of tumor and mesorectum (TMR) was developed based on selected radiomics features of three VOIs of the primary tumors and peritumoral mesorectum. Selected clinical and semantic factors and radiomics features of two VOIs of the primary tumors were used to develop a clinical-tumor radiomics model (CTR). Selected clinical and semantic factors and radiomics features of all three VOIs were used to develop a clinical-tumor and mesorectum radiomics model (CTMR).

The performance of the models in predicting LN status was first evaluated in the training cohort, then in the testing cohort by plotting a receiver operating characteristic (ROC) curve and calculating the area under the curve (AUC). The corresponding accuracy, sensitivity, specificity, negative predictive values (NPV), and positive predictive values (PPV) were then calculated. The differences in the AUCs of the five models were compared using DeLong’ test.

## Results

### Patient Characteristics

The demographic characteristics of patients in the training and testing cohorts are shown in **Table 1**. There were no significant differences between the two cohorts in LN prevalence (*P* = 0.892). LN metastasis positivity was 43.9 and 42.9% in the training and testing cohorts, respectively. The characteristics of the two cohorts did not differ significantly, which justifies their use as training and testing cohorts (*P* values ranged from 0.121 to 0.906). The maximum LN short diameter differed significantly between the pLN+ and pLN− groups in both cohorts (*P* = 0.001 and *P* = 0.004, respectively). The location of the primary tumor differed significantly between the pLN+ and pLN− groups in the training cohorts (*P* = 0.008). Good inter- and intra-observer reproducibility of tumor segmentation was achieved. The DSC for intra-observer agreement ranged from 0.793 to 0.865; for inter-observer agreement, it ranged from 0.773 to 0.847, which demonstrates good consistency.

**Table 1:** Characteristics of patients in training and testing cohorts.

Characteristic	Training cohort			Testing cohort			*P* ^※^
	**pLN+n = 54**	**pLN−n = 69**	***P***	**pLN+n = 27**	**pLN−n = 36**	***P***	**0.892**
Gender			0.090^a^			0.184^a^	0.906^a^
Male	33(61.1)	52(75.4)		16(59.3)	27(75.0)		
Female	21(38.9)	17(24.6)		11(40.7)	9(25.0)		
Age, years	60(53−67)	60(51.5−70.5)	0.520^c^	57.6 ± 12.7	59.3 ± 10.2	0.571^b^	0.388^b^
CEA level			0.508^a^			0.052^a^	0.324^a^
Normal	39(72.2)	46(66.7)		13(48.1)	26(72.2)		
Abnormal	15(27.8)	23(33.3)		14(51.9)	10(27.8)		
CA19-9 level			0.289^a^			0.643^a^	0.684^a^
Normal	47(87.0)	64(92.8)		24(88.9)	34(94.4)		
Abnormal	7(13.0)	5(7.2)		3(11.1)	2(5.6)		
Location of primary tumor			0.008^a^*			0.128^a^	0.397^a^
Upper	7(13.0)	1(1.4)		0(0)	1(2.8)		
Middle	29(53.7)	31(44.9)		17(63.0)	15(41.7)		
Lower	18(33.3)	37(53.6)		10(37.0)	20(55.6)		
Tumor length(cm)	5.2 ± 2.2	5.3 ± .3	0.858^b^	5.4 ± 2.0	4.8 ± 1.9	0.199^b^	0.594^b^
Tumor thickness(cm)	1.3(1.1−1.5)	1.3(1.1−1.6)	0.910^c^	1.1(0.9−1.6)	1.3(1.0−1.6)	0.512^c^	0.194^c^
Extramural depth of invasion(mm)	5.0(2.0−7.3)	4(0-6)	0.126^c^	5.0(3.0−8.0)	4.0(0.3−8.0)	0.212^c^	0.268^c^
Maximum LN short diameter(mm)	6.0(4.0−8.0)	6.0(3.0−6.0)	0.001^c^*	7.0(5.0-9.0)	5.0(3.3−6.8)	0.004^c^*	0.121^c^
Invasion of MRF			0.708^a^			0.504^a^	0.598^a^
Negative	43(79.6)	53(76.8)		19(70.4)	28(77.8)		
Positive	11(20.4)	16(23.2)		8(29.6)	8(22.2)		
MRI-reported lymph status			0.063^a^			0.059^a^	0.271^a^
Negative	14(25.9)	29(42)		4(14.8)	13(36.1)		
Positive	40(74.1)	40(58)		23(85.2)	23(63.9)		

pLN-, pathological N0 stage; pLN +, pathological N1-N2 stage; CEA, carcinoembryonic antigen; CA19-9, carbohydrate antigen 19-9; MRF, mesorectal fascia; a, Chi-square test or Fisher’s exact test, data are number of patients, with percentages in parentheses; b, Independent sample t test, data are mean ± SD; c, Mann-Whitney U test, data are median, with Interquartile range in parentheses.* p value <0.05; ※The comparison between the training cohort and testing cohort. The threshold value for CEA level was 5ng/mL and >5 ng/mL, and the threshold value of CA 19-9 level was 39 U/mL and >39 U/ml, according to the normal range used in clinics.

### Feature Selection and Model Construction

Selected features after Spearman’s rank correlation coefficient and LASSO regression and corresponding coefficients and the intercept of the constructed five SVM prediction models in the training cohort are shown in **Supplementary Tables 2 to 6**. The possibility of LN metastasis was calculated for each patient *via* a linear combination of selected features that were weighted by their respective coefficients in the SVM model and adding the intercept.

### Performance of the Models

**Figure 3:**
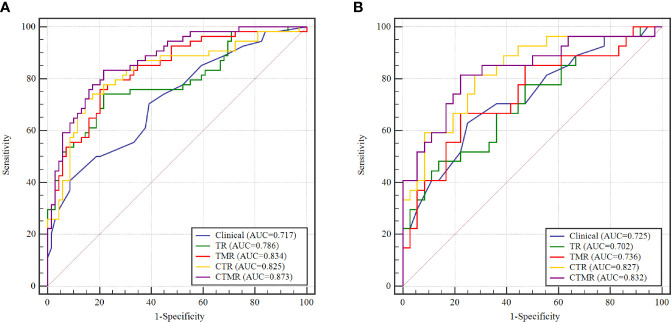
The receiver operator characteristic (ROC) curves to discriminate pLN+ from pLN− for the five models on the training cohort **(A)** and testing cohorts **(B)**. AUC, area under the curve; TR, radiomics model of tumor; TMR, radiomics model of tumor and mesorectum; CTR, clinical-tumor radiomics model; CTMR, clinical-tumor and mesorectum radiomics model.

The ROC curves and corresponding AUC values that distinguish between pLN+ and pLN− in the five models are shown in **Table 2** and **Figure 3**. The clinical model performed moderate when classifying between pLN+ and pLN−, with an AUC of 0.717 (95% confidence interval (CI), 0.629–0.795) and 0.725 (95% CI, 0.598–0.830) in the training and testing cohorts, respectively. There was no significant difference between the AUC of the clinical and single-regional TR models in the two cohorts (training: AUC = 0.786 (95% CI, 0.702–0.854), *P* = 0.222; testing: AUC = 0.702 (95% CI, 0.573–0.810), P = 0.801). Compared with the single-regional TR model, the multiregional-based CTMR model showed improved AUCs in the two cohorts (training: AUC = 0.837 (95% CI, 0.801–0.926), *P* = 0.009; testing: AUC = 0.832 (95% CI, 0.717–0.915), *P* = 0.030). The single-regional CTR model outperformed the TR model only in the testing cohort (AUC = 0.827 (95% CI, 0.711–0.911), *P* = 0.016). Compared with the multiregional TMR model, the CTMR model showed improved AUCs in the testing cohort (*P* = 0.015). The TMR, CTR, and CTMR models outperformed the clinical model only in the training cohort (*P* values ranged from <0.001 to 0.014), while no significant differences were seen in the testing cohort.

**Table 2:** The detailed AUC vaues and *p* values among models on the training cohort and testing cohorts.

Cohorts	Model	AUC (95％CI)	*P*	*P1*	*P2*	*P3*	*P4*
Training	Clinical	0.717(0.629–0.795)	<0.001^*^				
	TR	0.786(0.702–0.854)	<0.001^*^	0.222			
	TMR	0.834(0.756–0.895)	<0.001^*^	0.014^*^	0.106		
	CTR	0.825(0.746–0.888)	<0.001^*^	0.003^*^	0.198	0.749	
	CTMR	0.873(0.801–0.926)	<0.001^*^	<0.001^*^	0.009^*^	0.132	0.043^*^
Testing	Clinical	0.725(0.598–0.830)	<0.001^*^				
	TR	0.702(0.573–0.810)	0.003^*^	0.801			
	TMR	0.736(0.609–0.839)	<0.001^*^	0.903	0.486		
	CTR	0.827(0.711–0.911)	<0.001^*^	0.061	0.016^*^	0.116	
	CTMR	0.832(0.717–0.915)	<0.001^*^	0.068	0.030^*^	0.015^*^	0.885

TR, radiomics model of tumor; TMR, the radiomics model of tumor and mesorectum; CTR, clinical-tumor radiomics model; CTMR, clinical-tumor and mesorectum radiomics model; AUC, the area under the curve; CI, confidence interval. ^*^P < 0.05; P1, p values between clinical model and other models; P2, p values between TR model and other models; P3, p values between TMR model and other models; P4, p values between CTR model and CTMR models. p values of P1 to P4 calculated using ROC test by Delong test.

**Table 3** summarizes the accuracy, sensitivity, specificity, PPV, and NPV of the five models in detail. The clinical model was able to discriminate between pLN+ and pLN− in the training and testing cohorts with an accuracy of 0.650 and 0.635 respectively. All the performance indexes of the TR model were better than those of the clinical model in the training cohort; in the testing cohort, only specificity was higher in the clinical model. When tumor features were combined with mesorectum features, the resulting TMR model showed an improved accuracy with values of 0.722 and 0.635 in the two cohorts, outperforming the clinical model in the training cohort and having the same accuracy as in the testing cohort. When the single-regional and multiregional radiomics models were combined with clinical factors, the resulting CTR and CTMR models had higher accuracies and better performance indexes than the uncombined models in the two cohorts. With the exception of the CTR and CTMR models, which had the same sensitivity in the testing cohort (0.815), the CTMR model had the highest performance indicators in the two cohorts.

**Table 3:** Predictive performances among models on the training cohort and testing cohorts.

Model	Cohorts	Accuracy (95％CI)	Sensitivity	Specificity	PPV	NPV
Clinical	Training	0.650(0.566–0.734)	0.704	0.609	0.585	0.724
	Testing	0.635(0.516–0.754)	0.704	0.583	0.559	0.724
TR	Training	0.707(0.627–0.787)	0.741	0.681	0.645	0.770
	Testing	0.619(0.499–0.739)	0.593	0.639	0.552	0.676
TMR	Training	0.772(0.698–0.846)	0.778	0.768	0.724	0.815
	Testing	0.635(0.616–0.754)	0.667	0.611	0.563	0.710
CTR	Training	0.764(0.689–0.839)	0.778	0.754	0.712	0.813
	Testing	0.746(0.639–0.853)	0.815	0.694	0.667	0.833
CTMR	Training	0.789(0.717–0.861)	0.796	0.783	0.741	0.831
	Testing	0.778(0.675–0.881)	0.815	0.750	0.710	0.844

The cutoff was 0 for all the models. TR, radiomics model of tumor; TMR, The radiomics model of tumor and mesorectum; CTR, clinical-tumor radiomics model; CTMR, clinical-tumor and mesorectum radiomics model; CI, confidence interval. PPV, positive predictive value; NPV, negative predictive value.

## Discussion

In this study, we explored the diagnostic value of multiple models which included clinical factors, single-regional radiomics, multiregional radiomics, and combinations of clinical and radiomics models based on MRI to preoperatively predict LN metastasis in patients with rectal cancer. Our results showed that the established models had good predictive performance, and a multifactorial model based on multiregional radiomics combined with clinical factors had better classification performance and diagnostic accuracy, suggesting that it can act as a relatively non-invasive auxiliary evaluation tool for clinical decision-making.

Preoperative LN staging in patients with rectal cancer remains a challenge for radiologists. Previous studies have reported the use of clinical and semantic factors such as CEA and serum angiopoietin-like protein 2 levels, histopathological features, the diameter of LN, and morphological features (22–25) to predict LN status in patients with rectal cancer. However, these features are not enough to reliably diagnose LN metastasis in patients with rectal cancer (2, 5, 25). In this study, we found that the maximum LN short diameter was significantly different between the pLN− and pLN+ patients in both the training and testing cohorts, with a bigger LN diameter indicating an increased probability of metastasis. Several previous studies have shown that some clinical characteristics were related to LN metastasis (3, 24). However, in our study, clinical characteristics such as CEA and CA19-9 had no additional value for predicting LN status. These results may be related to characteristics of the study population itself, such as the sample size. After feature selection, two semantic indicators, namely the maximum LN short diameter and tumor location, were included in the final clinical model. Our results also showed that a model based purely on semantic variables had relatively low sensitivity and specificity for the prediction of LN status, which may lead to moderate accuracy for diagnosis. However, this result should be interpreted with caution, as clinical variables vary from population to population.

At present, several studies have reported the role of radiomics in predicting LN metastasis in rectal cancer. In comparison, none of the rectal MRI studies had ever focused on peritumoral tissue and the microenvironment. Huang et al. (16) used an enhanced CT-based radiomics model to discriminate LN metastasis in colorectal cancer patients with a concordance index of 0.736–0.778. However, previous studies focused on both colonic and rectal lesions using CT data in regions of interest (ROIs) of the primary tumor region alone. In our study, the segmentation of images was performed layer by layer, and 3D VOIs were constructed. Previous studies have shown that 3D VOIs are more representative of the heterogeneity of the whole lesion than 2D ROIs (26). Moreover, the LN status of rectal cancer is important for clinical decision making. MRI is considered to be the optimal imaging modality for the primary staging of rectal cancer (2). Yang et al. used T2WI histogram features of the primary rectal tumor to predict the existence of LN metastasis with moderate-to-good diagnostic power and an AUC of 0.648 to 0.750 (17). Yang et al. segmented the single-regional ROIs of rectal cancer images to extract histogram features. Previous studies have indicated that multiregional MRI radiomics allows for a comprehensive characterization of the tumor heterogeneity (27, 28). In addition to the region of the tumor, the surrounding mesorectal tissues may also exhibit abnormal microscopic changes in the microvascular and lymphatic networks, the extracellular matrix, and the interstitial pressure, which should not be ignored (3, 29). A central hypothesis driving radiomics research is that radiomics has the potential to quantitatively measure intra- and intertumoral heterogeneity (11). When the current multiregional radiomics signature was introduced into the prediction model of rectal cancer, the performance improved when compared to that of the single-regional model (3). Hence, the radiomics model constructed in our study included the VOIs of the primary tumor and the mesorectum at the lesion level on the morphological T2WI sequence and the VOIs of the primary tumor on the functional DWI sequence. Our study found that the multiregional radiomics model showed minor non-significant improvements in AUC compared with a single-regional radiomics model (*P* = 0.486), but the former had better accuracy.

Considering the global nature of the model, clinical, treatment, and biological or genetic information should be included in the radiomics analysis process (12). Our results showed no significant difference in AUCs between the clinical, single-regional radiomics, and multiregional radiomics models, which showed that clinical models and radiomics models have similar predictive performance. The combination of clinical factors with single-regional and multiregional radiomics features improved the performance of the model, and the model with the combination of clinical factors and multiregional radiomics features had the highest AUC and accuracy values. This indicated that the clinical information in the combined models may contribute relatively more to the prediction performance than the radiomics features. So, clinical and semantic factors also play an important role in the prediction of LN metastasis of rectal cancer. The sensitivity and NPV of the combined models were high, indicating that the models can accurately identify true pLN+ and true pLN− patients. The need for a model to determine LN metastasis—one that can accurately identify patients who need neoadjuvant chemoradiotherapy—is high. For patients with tumors confined to T0 and T1 staging, accurate identification of pLN− patients may actually change clinical decision-making; that is, only local excision would be performed to avoid the pain caused by surgery, and it is possible for patients with lower-stage tumors to maintain anal sphincter function. Therefore, from a clinical perspective, the significance of accurately identifying pLN− patients is great, and we conclude that the addition of clinical factors to radiomics analysis potentially creates a substantial biomarker for assessing the risk of LN metastasis and could be applied in clinical practice.

Our study had several limitations. Firstly, the sample size was relatively small, and the retrospective study lacked independent external validation. In the future, our results should be prospectively validated in multicenter clinical trials. Secondly, genomic characteristics were not considered. Radiogenomics, which focuses on the relationship between imaging phenotypes and genomics, has emerged in the field of cancer research and has attracted increasing interest (29). Thirdly, manual segmentation was used in this study, which is time-consuming and error-prone. Therefore, a reliable and robust automatic segmentation tool is necessary to solve this problem.

## Conclusions

In conclusion, our findings demonstrated that multiregional-based radiomics features from multiparametric MRIs of patients with rectal cancer combined with clinical data can improve efficacy in non-invasively predicting LN metastasis and could serve as a useful tool to preoperatively guide individualized surgical decision-making of patients with rectal cancer.

## Data Availability Statement

The original contributions presented in the study are included in the article/**Supplementary Material**; further inquiries can be directed to the corresponding authors.

## Ethics Statement

The studies involving human participants were reviewed and approved by The First Hospital of Jilin University. Written informed consent for participation was not required for this study in accordance with the national legislation and the institutional requirements.

## Author Contributions

HZ, YF, and XL contributed to the conception and design. XL, YX, and YZ organized the database. QY, KH, and CZ administrated, managed patients, provided technical support, etc. XL wrote the first draft of the manuscript. XL and JS performed the statistical analysis. YF, JS, and HZ reviewed and revised the manuscript. All authors contributed to the article and approved the submitted version.

## Funding

This study was supported by the Jilin Province Science and Technology Department Science and Technology Innovation Talents Cultivation Program (20180519008JH), Jilin Provincial Department of Finance (98022740001, 2018SCZWSZX-026, JLSCZD2019-062), Jilin Province Development and Reform Commission (2017C020), Jilin Province Health and Family Planning Commission (2017J073), and Prevention and Control of Major Diseases Science and Technology Action Plan of China (ZX-07-C2016003).

## Conflict of Interest

JS was employed by Philips Investment Co. Ltd.

The remaining authors declare that the research was conducted in the absence of any commercial or financial relationships that could be construed as a potential conflict of interest.
