# Supplementary material for: Multiregional-Based Magnetic Resonance Imaging Radiomics Combined With Clinical Data Improves Efficacy in Predicting Lymph Node Metastasis of Rectal Cancer
Source: Front Oncol. 2021 Feb 18;10:585767. doi: 10.3389/fonc.2020.585767 (PMC7930475; doi:10.3389/fonc.2020.585767)
Supplement: Supplementary file 1 [file DataSheet_1.docx]

Supplementary Material

# Supplementary Tables

## Supplementary Table 1

**Supplementary Table 1.** The Extracted Features by Philips Radiomics software Using PyRadiomics

| **Indexes** | **Introduction** | **Feature number** |
| --- | --- | --- |
| Direct features | Including first-order statistics features, shape-based features, gray level co-occurrence matrix features, gray level size zone matrix features, gray level run length matrix features, neigbouring gray tone difference matrix features and gray level dependence matrix features | 105 |
| Indirect features | Calculated based on direct features, through the algorithm of square, squareroot, logarithm and exponential | 368 |
| Wavelets transform features | Information about the frequency of similar SIs and describes the wavelet transform of the pixels in the ROI | 720 |
| Laplacian of Gaussian filtered features | Description of texture based on the images filtered by Laplacian of Gaussian | 460 |

ROI =regions of interest

## Supplementary Table 2

**Supplementary Table 2.** The Final Selected Features and Corresponding Coefficients and Intercept in Clinical Model

| **Features source** | **Features and Intercept** | **Coefficients** |
| --- | --- | --- |
| Clinical -semantic | Maximum lymph node short diameter | 1.418 |
| Clinical -semantic | Location of primary tumor | -0.698 |
|  | Intercept | -0.029 |

## Supplementary Table 3

**Supplementary Table 3.** The Final Selected Features and Corresponding Coefficients and Intercept in TR Model

| **Features source** | **Features and Intercept** | **Coefficients** |
| --- | --- | --- |
| Radiomics- tumor-T2WI | WaveletNGTDM_wavelet-HLL_Strength | 0.901 |
| Radiomics -tumor-T2WI | ShapeBased_SphericalDisproportion | 0.778 |
| Radiomics -tumor-T2WI | WaveletGLCM_wavelet-HLL_Idn | 0.596 |
| Radiomics -tumor-T2WI | WaveletGLDM_wavelet-HLL_LargeDependenceLowGrayLevelEmphasis | 0.519 |
| Radiomics -tumor-T2WI | WaveletFirstOrder_wavelet-LLH_Kurtosis | 0.510 |
| Radiomics -tumor-T2WI | WaveletGLCM_wavelet-LHL_Idn | 0.433 |
| Radiomics -tumor-T2WI | WaveletGLCM_wavelet-LHH_Correlation | 0.416 |
| Radiomics -tumor-T2WI | WaveletFirstOrder_wavelet-HLL_Kurtosis | 0.165 |
| Radiomics -tumor-T2WI | WaveletFirstOrder_wavelet-HLL_Skewness | -0.439 |
| Radiomics -tumor-T2WI | GLCM_MCC | -0.867 |
| Radiomics -tumor-T2WI | ExponentialGLRLM_exponential_RunEntropy | -0.928 |
|  | Intercept | -0.447 |

TR = radiomics model of tumor, T2WI = T2-weighted imaging, NGTDM = [Neighbouring Gray Tone Difference Matrix](https://pyradiomics.readthedocs.io/en/latest/features.html#radiomics.ngtdm.RadiomicsNGTDM), GLCM = [Gray Level Cooccurence Matrix](https://pyradiomics.readthedocs.io/en/latest/features.html#radiomics.glcm.RadiomicsGLCM), GLDM = [Gray Level Dependence Matrix](https://pyradiomics.readthedocs.io/en/latest/features.html#radiomics.gldm.RadiomicsGLDM), GLSZM = [Gray Level Size Zone Matrix](https://pyradiomics.readthedocs.io/en/latest/features.html#radiomics.glszm.RadiomicsGLSZM), GLRLM = [Gray Level Run Length Matrix](https://pyradiomics.readthedocs.io/en/latest/features.html#radiomics.glrlm.RadiomicsGLRLM)

## Supplementary Table 4

**Supplementary Table 4.** The Final Selected Features and Corresponding Coefficients and Intercept in TMR Model

| **Features source** | **Features and Intercept** | **Coefficients** |
| --- | --- | --- |
| Radiomics -tumor-T2WI | ExponentialGLRLM_exponential_RunEntropy | -1.473 |
| Radiomics -tumor-T2WI | WaveletGLCM_wavelet-HLL_Idn | 0.732 |
| Radiomics -tumor-T2WI | WaveletNGTDM_wavelet-HLL_Strength | 0.716 |
| Radiomics -tumor-T2WI | ShapeBased_SphericalDisproportion | 0.472 |
| Radiomics -tumor-T2WI | WaveletFirstOrder_wavelet-LLH_Kurtosis | 0.404 |
| Radiomics -tumor-T2WI | WaveletGLCM_wavelet-LHL_Idn | 0.396 |
| Radiomics -tumor-T2WI | WaveletGLCM_wavelet-HLL_JointAverage | 0.320 |
| Radiomics -tumor-T2WI | WaveletGLCM_wavelet-LHH_Correlation | 0.211 |
| Radiomics -tumor-T2WI | GLCM_MCC | -0.833 |
| Radiomics -mesorectum-T2WI | ExponentialGLSZM_exponential_ZoneEntropy | -0.903 |
| Radiomics - mesorectum -T2WI | ExponentialGLCM_exponential_Imc2 | -0.960 |
| Radiomics - mesorectum -T2WI | ShapeBased_Flatness | 1.254 |
| Radiomics - mesorectum -T2WI | ExponentialGLDM_exponential_SmallDependenceHighGrayLevelEmphasis | 1.136 |
|  | Intercept | 0.275 |

TMR = The radiomics model of tumor and mesorectum, T2WI = T2-weighted imaging, GLRLM = [Gray Level Run Length Matrix](https://pyradiomics.readthedocs.io/en/latest/features.html#radiomics.glrlm.RadiomicsGLRLM), GLCM = [Gray Level Cooccurence Matrix](https://pyradiomics.readthedocs.io/en/latest/features.html#radiomics.glcm.RadiomicsGLCM), NGTDM = [Neighbouring Gray Tone Difference Matrix](https://pyradiomics.readthedocs.io/en/latest/features.html#radiomics.ngtdm.RadiomicsNGTDM), GLSZM = [Gray Level Size Zone Matrix](https://pyradiomics.readthedocs.io/en/latest/features.html#radiomics.glszm.RadiomicsGLSZM), GLDM = Gray Level Dependence Matrix

## Supplementary Table 5

**Supplementary Table 5.** The Final Selected Features and Corresponding Coefficients and Intercept in CTR Model

| **Features source** | **Features and Intercept** | **Coefficients** |
| --- | --- | --- |
| Clinical -semantic | Maximum lymph node short diameter | 1.088 |
| Clinical -semantic | Location of primary tumor | -0.503 |
| Radiomics -tumor-T2WI | WaveletNGTDM_wavelet-HLL_Strength | 0.822 |
| Radiomics -tumor-T2WI | WaveletFirstOrder_wavelet-LLH_Kurtosis | 0.529 |
| Radiomics -tumor-T2WI | WaveletGLCM_wavelet-LHH_Correlation | 0.525 |
| Radiomics -tumor-T2WI | WaveletFirstOrder_wavelet-HLL_Kurtosis | 0.420 |
| Radiomics -tumor-T2WI | ShapeBased_SphericalDisproportion | 0.396 |
| Radiomics -tumor-T2WI | WaveletGLCM_wavelet-LHL_Idn | 0.268 |
| Radiomics -tumor-T2WI | WaveletGLCM_wavelet-HLL_Idn | 0.199 |
| Radiomics -tumor-T2WI | WaveletFirstOrder_wavelet-HLL_Skewness | -0.345 |
| Radiomics -tumor-T2WI | ExponentialGLRLM_exponential_RunEntropy | -0.733 |
| Radiomics -tumor-T2WI | GLCM_MCC | -0.799 |
|  | Intercept | -0.220 |

CTR = clinical-tumor radiomics model, T2WI = T2-weighted imaging, NGTDM = [Neighbouring Gray Tone Difference Matrix](https://pyradiomics.readthedocs.io/en/latest/features.html#radiomics.ngtdm.RadiomicsNGTDM), GLCM = [Gray Level Cooccurence Matrix](https://pyradiomics.readthedocs.io/en/latest/features.html#radiomics.glcm.RadiomicsGLCM), GLRLM = Gray Level Run Length Matrix

## Supplementary Table 6

**Supplementary Table 6.** The Final Selected Features and Corresponding Coefficients and Intercept in CTMR Model

| **Features source** | **Features and Intercept** | **Coefficients** |
| --- | --- | --- |
| Clinical -semantic | Maximum lymph node short diameter | 1.208 |
| Clinical -semantic | Location of primary tumor | -0.192 |
| Radiomics -tumor-T2WI | ExponentialGLRLM_exponential_RunEntropy | -1.298 |
| Radiomics -tumor-T2WI | WaveletGLCM_wavelet-HLL_Idn | 0.677 |
| Radiomics -tumor-T2WI | WaveletNGTDM_wavelet-HLL_Strength | 0.664 |
| Radiomics -tumor-T2WI | WaveletFirstOrder_wavelet-LLH_Kurtosis | 0.571 |
| Radiomics -tumor-T2WI | WaveletFirstOrder_wavelet-HLL_Skewness | -0.549 |
| Radiomics -tumor-T2WI | GLCM_MCC | -0.786 |
| Radiomics -mesorectum-T2WI | ExponentialGLCM_exponential_Imc2 | -0.838 |
| Radiomics -mesorectum-T2WI | ExponentialGLSZM_exponential_ZoneEntropy | -0.868 |
| Radiomics -mesorectum-T2WI | ExponentialNGTDM_exponential_Busyness | -0.880 |
| Radiomics -mesorectum-T2WI | ShapeBased_Flatness | 1.545 |
| Radiomics-mesorectum-T2WI | ExponentialGLDM_exponential_SmallDependenceHighGrayLevelEmphasis | 1.224 |
|  | Intercept | 0.725 |

CTMR = clinical-tumor and mesorectum radiomics model, T2WI = T2-weighted imaging, GLRLM = [Gray Level Run Length Matrix](https://pyradiomics.readthedocs.io/en/latest/features.html#radiomics.glrlm.RadiomicsGLRLM), GLCM = [Gray Level Cooccurence Matrix](https://pyradiomics.readthedocs.io/en/latest/features.html#radiomics.glcm.RadiomicsGLCM), NGTDM = [Neighbouring Gray Tone Difference Matrix](https://pyradiomics.readthedocs.io/en/latest/features.html#radiomics.ngtdm.RadiomicsNGTDM), GLSZM = [Gray Level Size Zone Matrix](https://pyradiomics.readthedocs.io/en/latest/features.html#radiomics.glszm.RadiomicsGLSZM), GLDM = Gray Level Dependence Matrix
